# Gastric cancer: an overview

**DOI:** 10.1590/1806-9282.2024S116

**Published:** 2024-06-07

**Authors:** Gardênia Costa do Carmo, Rafaelle Marques Cavalcante, Tarson Maia Furtado de Aquino

**Affiliations:** 1 Fortaleza General Hospital, Gastroenterology Service – Fortaleza (CE), Brazil.

## INTRODUCTION

Gastric cancer (GC) represents an important global health problem since it is the fifth leading cancer in the world and the third leading cause of cancer-related death^1^, although the overall incidence is declining. This decline has been mainly attributed to the decreased prevalence of *Helicobacter pylori* (*Hp*) infection, but also to the progress in food storage and preservation, probably by allowing the reduction of salty and smoked food consumption^2^. There is great geographic variation in GC incidence, with the majority of new diagnoses per year of GC occurring mainly in Asian and South American countries^3^. In Brazil, it is the third most common type among men and the fifth among women^4^. GC occurs approximately twice as frequently in men as in women, with most cases occurring after the age of 60 years^1^. Adenocarcinoma is the most common histological type, accountable for about 90–95% of cases^5^.

There are two main topographic subsites of GC: esophagogastric junction (EGJ) and nonjunctional. The descriptive epidemiology and risk factor profiles of each are different. In contrast to the pattern seen with nonjunctional GC, the incidence rates of adenocarcinomas at the EGJ are rising^6^, probably due to an increased rate of obesity and gastroesophageal reflux disease (GERD), which are considered the major risk factors for the latter^7^. Furthermore, current data suggests an increase in the incidence of nonjunctional GC in a group of young individuals, especially women under the age of 50 years^8^.

## ETIOLOGY AND PATHOGENESIS

Numerous dietary, environmental, and genetic risk factors have been related to gastric adenocarcinoma. The dominant risk factor remains, however, *Hp* infection and the associated chronic-active inflammation of the gastric mucosa. Up to 10% of GCs can be attributed to less common causes, including infection with the Epstein-Barr virus (EBV), autoimmune gastritis, and Menetrier's disease. Other factors associated with increased risk include tobacco smoking, low socioeconomic status, low level of physical activity, and radiation exposure; obesity and GERD are only associated with increased risk of EGJ GC^6^. Although most GC are sporadic, familial clustering is observed in up to 10% of patients^9^.

Gastric cancer can be subdivided using the Laurén classification into distinct histologic subtypes with different epidemiologic and prognostic features. Well-differentiated (intestinal) GC is predominately found in individuals of an older age, >70 years, who are mostly male and patients present with larger tumor sizes. This subtype has overall better prognoses than the poorly differentiated (diffuse) subtype. The diffuse subtype has poor survival statistics and is commonly found in younger women^10^. Extensive involvement of the stomach by that subtype can result in a rigid and thickened stomach, a condition referred to as linitis plastic. Another key feature of diffuse subtype cancers are signet-ring cells, special mucin-filled cells that are not present in intestinal subtype adenocarcinomas. There are also mixed phenotypes that contain heterogeneous areas that feature predominantly either intestinal or diffuse characteristics. The mixed subtype is present within a much smaller subset of patients, usually male, and it is known to be highly invasive and metastatic^6^.

It is accepted that the development of intestinal subtype GC occurs through a multistep process in which the normal mucosa is sequentially transformed into a hyperproliferative epithelium, followed by metaplastic processes leading to preneoplastic conditions (glandular atrophy, intestinal metaplasia), dysplasia, and then carcinoma^11^. Correa et al., postulates that there is a temporal sequence of preneoplastic changes that eventually lead to the development of GC. A common feature of the initiation and progression to intestinal subtype GC is chronic inflammation of the gastric mucosa by *Hp* infection^12^. Eradication of *Hp* has the potential to prevent GC as shown in recent meta-analyses, particularly if there are no preneoplastic conditions of the gastric mucosa at the time of intervention^13^.

## SCREENING

An important question is whether there is room for a population-based screening for GC. While it is justified and already adopted in several Asian countries where the GC incidence is high, it is much more debatable in the countries with low incidence. Guidelines from high-risk areas recommend biennial GC screening via upper endoscopy or upper gastrointestinal series for men and women aged ≥40 years^14^.

Patients with atrophic gastritis (AG) and gastric intestinal metaplasia (GIM) should be tested for *Hp* infection and, if positive, should be eradicated. Guidelines recommend endoscopic surveillance every 3 years in patients in whom extensive AG and/or extensive incomplete GIM has been diagnosed^15,16^.

## CLINICAL MANIFESTATIONS

The diagnosis of GC is generally made when the patient undergoes an endoscopy due to dyspeptic or reflux complaints. In more advanced cases, the individual may experience anemia, gastrointestinal bleeding, vomiting, weight loss or dysphagia^17^. The most common symptom related to the worst outcome is cachexia^18^.

Paraneoplastic syndromes are a rare manifestation of GCs. These include dermatological findings such as acanthosis nigricans, membranous nephropathy, microangiopathic hemolytic anemia, and trousseau syndrome (hypercoagulable state)^19^. Although a strong relationship between GC and SIADH (syndrome of inappropriate antidiuretic hormone) secretion has not yet been established, it is suggested that it can be included as a differential diagnosis associated with SIADH^20^. Hypercalcemia is extremely rare in metastatic gastric adenocarcinoma^21^.

## EXAMS FOR DIAGNOSIS AND STAGING

### Upper digestive endoscopy with biopsy

Fundamental exam for diagnosis, staging, treatment, and palliative resection^22^ enables the identification of preneoplastic and early lesions, which are suspected in the presence of surface irregularities or mucosal color^23^. Good representation of the material can be guaranteed by collecting 5–8 fragments^24^. A good exam must contain information about location, size, extension, infiltration, distance from the esophagogastric transition, and the pylorus, detailing the biopsies’ locations. In cases of high suspicion and repeated negative biopsies, including macrobiopsies, endoscopic or surgical resection should be considered^5^.

### Computed tomography of the chest and abdomen with oral and intravenous contrast

It must be performed after diagnosis for staging. Pelvis imaging can be performed only if there is clinical suspicion of involvement. When tomography is not possible, magnetic resonance imaging can be performed^22^.

### Echoendoscopy

Patients who do not present distant metastases or have lymph node (LN) involvement on initial tomography may undergo endoscopic ultrasound^22^. This examination will evaluate the extent of tumor invasion and determine the presence of abnormal or enlarged regional LN and the presence of ascites and metastases in nearby organs. It can also be used when there is doubt about the early appearance of the neoplasia^5^.

### Laparoscopy

It is an option for those who are not candidates for neoadjuvant therapy. This is a highly sensitive procedure for detecting peritoneal metastases or involvement of the gastric serosa, in addition to allowing cytology studies of the peritoneal fluid. If this is positive, the disease is considered metastatic even in the absence of visible implants^22^.

### Positron emission tomography/computed tomography

Positron emission tomography/computed tomography is not routinely recommended, but can be used to exclude metastatic disease when other diagnostic methods fail^22^. It has a limited role in the assessment of T stage, due to its low level of spatial resolution, but it could help in the detection of distant LN and bone metastasis^5^.

### Tumor markers

Analysis of tumor markers CA 19.9, CEA, CA 72.4 must be performed in all cases. Such markers have good sensitivity for recurrence, especially if elevated at the time of diagnosis. Your analysis must be carried out in a combined manner. Only CA 72.4 positivity should be considered as a specific indicator of cancer recurrence throughout the follow-up^5^.

## HISTOPATHOLOGICAL CLASSIFICATION AND STAGING

In addition to the previously mentioned Laurén histological classification, the tumor can be classified as grades I, II, and III, based on well, moderately, and poorly differentiated cells, respectively^22^.

According to the American Joint Committee on Cancer (AJCC)/Union for International Cancer Control (UICC) TNM (tumor-node-metastasis) 8th edition staging manual, tumors involving the EGJ that have an epicenter within 2 cm proximal to the gastric cardia or proximal stomach should be classified as esophageal cancer. Tumors with an epicenter located more than 2 cm distal from the EGJ, regardless of its involvement, should be classified as GC according to TNM parameters^25-27^.

The TNM classification correlates with 5-year survival and its clinical staging is shown in Table 1 ^25,26^. Regardless of the histological variant, the degree of invasion into the gastric wall determines the primary stage of the tumor. Early GC is defined as a lesion confined to the mucosa and submucosa (T1), regardless of LN involvement^23^. When it involves the muscularis propria, it is classified as T2, and T3 if the subserosa is affected. It is denominated as T4a in case that the tumor perforates the serosa and T4b if it invades adjacent structures^25,26^.

**Table 1:** Tumor-node-metastasis clinical staging of gastric cancer according to the American Joint Committee on Cancer/Union for International Cancer Control 8th edition.

Clinical stages			
Stage I	T1, T2	N0	M0
Stage IIA	T1, T2	N1, N2, N3	M0
Stage IIB	T3, T4a	N0	M0
Stage III	T3, T4a	N1, N2, N3	M0
Stage IVA	T4b	Any N	M0
Stage IVB	Any T	Any N	M1

It is recommended that a minimum number of 16 LNs is evaluated by the pathologist to improve the N staging accuracy. The number of regional LN with metastasis determines the N stage (N1: 1-2; N2: 3-6; and N3: 7 or more). The presence of distant metastasis is classified as M1^25-27^.

## TREATMENT

Multidisciplinary treatment is required, and the team must include gastroenterologists, surgeons, oncologists, radiologists, pathologists, nutritionists, endoscopists, and several other specialists. Combined modality therapy is generally used and more effective for patients with GC^17^.

Regular follow-up is recommended, tailored to each patient and stage of disease, for investigation and treatment of symptoms, provision of psychological support, and early detection of recurrence. Special attention must be paid to vitamin and mineral deficiencies, providing dietary support to the patient^24^.

**Figure 1:**
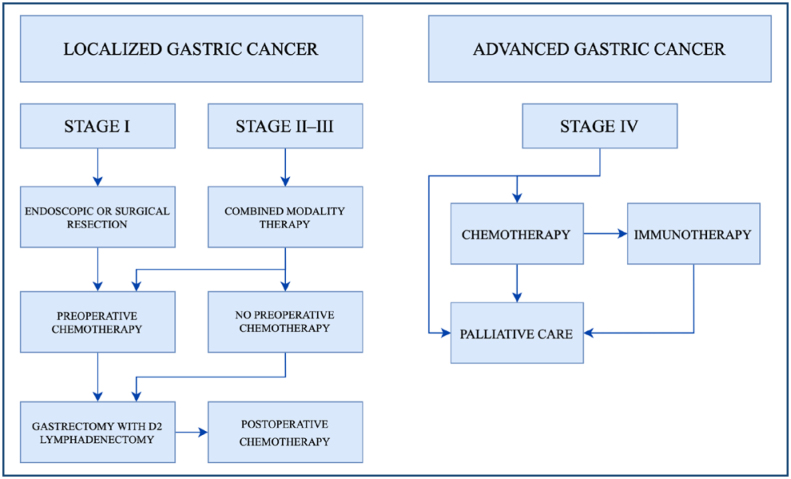
Treatment flowchart for gastric cancer.

A treatment flowchart for localized (stages I–III) and advanced (stage IV) GC is shown on Figure 1.

### Endoscopic treatment

Most early gastric tumors (neoplasms limited to the mucosa or submucosa) do not present LN metastasis, making the curative treatment of these lesions possible by endoscopy^22^.

Mucosectomy or endoscopic submucosal dissection (ESD) is indicated if: well to moderately differentiated tumor histology, size ≤2 cm, without invasion of the deep submucosa, non-ulcerated, and without lymphovascular invasion. Clear negative lateral and deep margins must be obtained^17^.

Gastric echoendoscopy can be performed before the procedure in order to assess the depth of tumor invasion^22^.

### Surgical resection

Patients with the absence of distant metastases should be considered for surgery with curative intent unless candidates present criteria for endoscopic resection. Gastrectomy (subtotal or total) with D2 lymphadenectomy is generally the surgery of choice^22^.

In advanced or metastatic cases, palliative surgery remains an alternative to cases of obstruction, perforation, or bleeding. Resection of metastases might be considered an individual approach in highly selected patients^22^.

### Chemotherapy

The preferential regimen depends on individual patient factors (using parameters such as performance status, age, comorbidities, and clinical contraindications), as well as clinical and surgical staging. Schemes like FLOT (5-fluorouracil–leucovorin–oxaliplatin–docetaxel), FOLFOX (5-fluorouracil–leucovorin– oxaliplatin), and CAPOX (capecitabine–oxaliplatin) can be prescribed^22^.

Perioperative chemotherapy (before and after surgery) or postoperative chemotherapy plus chemoradiation is listed as a preferred approach in current guidelines, although postoperative chemotherapy alone is an option after an adequate LN dissection^22^.

Patients in good clinical condition with metastatic disease have an indication for palliative chemotherapy^5^.

### Immunotherapy

Molecular targeted drugs are also present in the treatment of GC. Trastuzumab, a monoclonal antibody anti-human epidermal growth factor 2 (HER2) receptor, can be used for patients with HER2 overexpression^22^. Ramucirumab is another type of monoclonal antibody that binds to a different protein, i.e., vascular endothelial growth factor receptor 2 (VEGFR2), blocking receptor activation^17^.

Immune checkpoint blockade includes monoclonal antibodies that inhibit programmed cell death protein 1 (PD-1), programmed cell death ligand 1 (PD-L1), and cytotoxic T-lymphocyte antigen 4 (CTLA-4). This kind of therapy can be used in patients with advanced or metastatic GC^17^.

### Radiotherapy

Radiotherapy is recommended in some cases, such as those with an indication for adjuvant chemotherapy who did not have an adequate LN dissection during surgery^5^.

### Palliative care

Best supportive care must be offered for those patients with metastatic GC who have not responded to palliative chemotherapy or in poor clinical condition^5^.
